# Impact of operational effectiveness of long-lasting insecticidal nets (LLINs) on malaria transmission in pyrethroid-resistant areas

**DOI:** 10.1186/1756-3305-6-319

**Published:** 2013-11-04

**Authors:** Arthur Sovi, Roseric Azondékon, Rock Y Aïkpon, Renaud Govoétchan, Filémon Tokponnon, Fiacre Agossa, Albert S Salako, Frédéric Oké-Agbo, Bruno Aholoukpè, Mariam Okè, Dina Gbénou, Achille Massougbodji, Martin Akogbéto

**Affiliations:** 1 Centre de Recherche Entomologique de Cotonou, Cotonou 06 BP 2604, Benin; 2 Faculté des Sciences et Techniques, Université d’Abomey Calavi, Calavi, Bénin; 3 Programme Nationale de Lutte contre le Paludisme, Cotonou, Benin; 4 University of Massachusetts Amherst, Amherst, USA; 5 Faculté des Sciences de la Santé de l’Université d’Abomey- Calavi, Cotonou, Benin

**Keywords:** Resistance, LLINs, *An. gambiae*, Impact, Transmission, Behavior, Benin

## Abstract

**Background:**

A dynamic study on the transmission of malaria was conducted in two areas (R^+^ area: Low resistance area; R^+++^ area: High resistance area) in the department of Plateau in South Eastern Benin, where the population is protected by Long Lasting Insecticidal Nets (LLINs). The aim of this study was to determine if the resistance of malaria vectors to insecticides has an impact on their behavior and on the effectiveness of LLINs in the reduction of malaria transmission.

**Methods:**

Populations of *Anopheles gambiae s.l.* were sampled monthly by human landing catch in the two areas to evaluate human biting rates (HBR). Collected mosquitoes were identified morphologically and female *Anopheles* mosquitoes were tested for the presence of *Plasmodium falciparum* antigen as assessed using ELISA. The entomological inoculation rate (EIR) was also calculated (EIR = HBR x sporozoitic index [S]). We estimated the parity rate by dissecting the females of *An. gambiae*. Finally, window catch and spray catch were conducted in order to assess the blood feeding rate and the exophily rate of vectors.

**Results:**

After 6 months of tracking the mosquito's behavior in contact with the LLINs (Olyset) in R^+++^ and R^+^ areas, the entomological indicators of the transmission of malaria (parity rate and sporozoitic index) were similar in the two areas. Also, *An. gambiae* populations showed the same susceptibility to *P. falciparum* in both R^+^ and R^+++^ areas. The EIR and the exophily rate are higher in R^+^ area than in R^+++^ area. But the blood-feeding rate is lower in R^+^ area comparing to R^+++^.

**Conclusion:**

The highest entomological inoculation rate observed in R^+^ area is mostly due to the strong aggressive density of *An. gambiae* recorded in one of the study localities. On the other hand, the highest exophily rate and the low blood-feeding rate recorded in R^+^ area compared to R^+++^ area are not due to the resistance status of *An. gambiae*, but due to the differences in distribution and availability of breeding sites for *Anopheles* mosquitoes between areas. However, this phenomenon is not related to the resistance status, but is related to the environment instead.

## Background

Long Lasting Insecticidal nets (LLINs) are important tools in malaria vector control. For some years now, National Malaria Control Programs (NMCPs) have opted for a universal coverage and access of the populations to these impregnated materials.

Lengeler C [1] and O’Meara *et al*. [2] showed that the use of LLINs constitutes the most advantageous intervention in terms of cost-efficacy at a large scale. These LLINs not only represent a physical barrier in reducing contact between human and vector but also a chemical barrier. The chemical barrier acts on the mosquitoes through the deterrent, lethal and repellent effects. Thus, LLINs reduce the density, the frequency of blood feeding, the success of blood feeding and the survival of *Anopheles* vectors [3,4]. Moreover, the assets of this tool are mainly rooted in the fact that those protected by LLINs are no more exposed to the bites of *Anopheles* vectors, and a strong coverage rate also provides protection to the rest of the community [5-7]. Further, several results from studies carried out in Africa and in Papua New Guinea indicate the presence of an advantageous effect of LLINs at the community level. Indeed, LLINs have contributed to the reduction of the intensity of malaria transmission [8,9], the number of severe malaria cases [10] and infant mortality rates [5].

In July 2011, in order to ensure total coverage of the population, the NMCP in Benin increased their coverage by a large-scale distribution of LLINs (Olyset).

Unfortunately, a major problem with the use of LLINs currently is the appearance of the resistance of malaria vectors to insecticides, especially to pyrethroids. During the past few years, resistance to insecticides has become widespread in Western [11-15], Eastern [16], Central [17] and in Southern Africa [18]. Therefore, it is important for NMCPs to know if they should continue to promote LLINs. The resistance of *Anopheles* to insecticides was to some extent explored by N’guessan *et al*. [19] who demonstrated a reduction in efficacy of insecticide impregnated nets and Indoor Residual Sprays (IRS) with lambda-cyhalothrin in experimental huts in Benin. As these results seem worrying, they need to be further explored since the study was conducted in experimental huts, and therefore making it difficult to extrapolate what will happen at the community level. Thus, we conducted and implemented a study in natural settings, in a department of more than 200,000 people where LLINs were massively distributed. As a possible approach, the impact of these LLINs on the malaria transmission might be measured in two areas: one area where *An. gambiae* population is resistant to pyrethroids and one area where this species is susceptible to pyrethroids (control area). Due to the absence of a real area of susceptibility of *An. gambiae* to pyrethroids in Benin (Djègbè, personal communication), this second area was redefined and replaced by an area of low resistance status to pyrethroids, which we called “R^+^ area” as opposed to an area of high resistance status that we called “R^+++^ area”. These two areas were identified based on baseline resistance data collected in the department of Plateau (Djègbè, personal communication). The main goal of the current study was to assess the impact of two resistance levels on the operational effectiveness of LLINs. The entomological indicators of malaria transmission (Dynamic of *Anopheles* population, sporozoitic index, EIR, parity, exophily and blood feeding rate) were compared between the R^+^ and R^+++^ areas.

## Methods

### Study area

**Figure 1:**
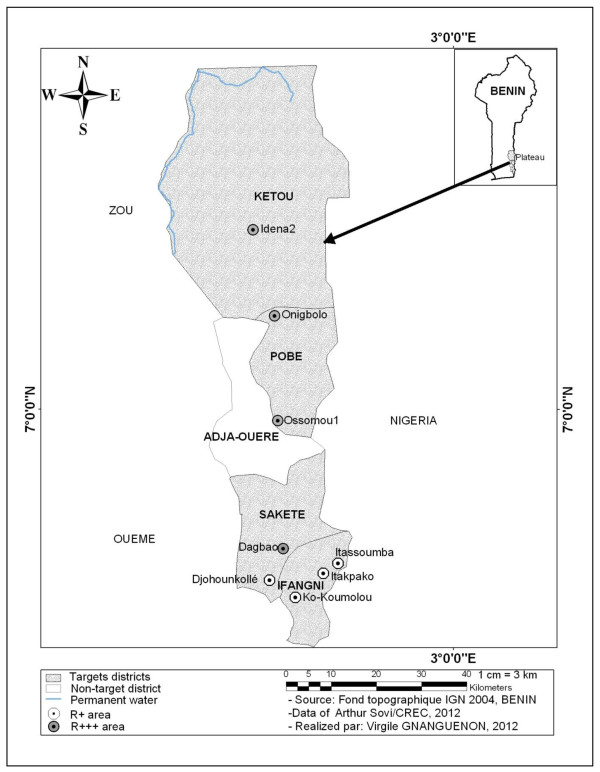
**Map showing the R**^**+ **^**and R**^**+++ **^**areas.**

The study was carried out in eight localities, which were chosen according to the following criteria: susceptibility level of malaria vectors to insecticides after using deltamethrin (0.05%) treated filter papers and the accessibility to the localities (Figure 1). The localities belonging to the R^+++^ area were defined as localities where the mortality rate of mosquitoes was less than 80%. Above this rate, the localities are considered as an R^+^ area (Djègbè, personal communication). Thus, the localities of Dagbao located in the district of Sakété, Onigbolo and Ossomou 1 in the district of Pobè and Idena 2 in the district of Kétou were classified as R^+++^ area. R^+^ localities included areas of Itakpako, Itassoumba and Ko-koumolou located in the district of Ifangni and Djohounkollé in the district of Sakété. In June 2012, a cross-sectional survey revealed that the prevalence of malaria in children under five years was 55%, 57.5%, 15%, 15%, 7.5%, 37.5% and 12.5% respectively at Itakpako, Itassoumba, Ko-Koumolou, Djohounkollé , Dagbao, Ossomou 1 and Idena 2 (Tokponnon, personal communication).

Overall, the study localities had similar ecological patterns except for the locality of Itassoumba, which has major environmental modification characterized by the presence of a market-gardening perimeter with several fishpond basins. This perimeter covers a surface of at least 4 hectares. More than a hundred basins are on this perimeter where tilapias and to a lesser extent catfishes are bred. In Itassoumba, fish farming occurs throughout the year. Off-season production of vegetables is also cultivated on the market-gardens and the fishpond area of Itassoumba from December to February corresponding to the dry season where vegetables are generally not available on the market.

### Entomological monitoring

For the measurement of the malaria transmission level in each locality, we collected *Anopheles* mosquitoes. These collections enabled us to evaluate the biting rate and the frequency of infected biting for each locality. In each locality, mosquitoes were collected using human landing catch inside and outside of two dwellings. The captures were made from 9:00 pm to 5:00 am. Two successive human landing catches were carried out per month in each locality for a total of 64 captures per night per month for all 8 localities.

Collected mosquitoes were identified in the morning to genus and species level using the morphological determination key by Gillies and de Meillon [20]. The vectors were dissected to determine their parity rate. Heads and thoraxes of the dissected mosquitoes were preserved on silicagel for the detection of *P. falciparum* using circumsporozoite protein (CSP) ELISA techniques [21,22].

From the mosquitoes captured and from the ELISA results, we determined the number of bites per man per night for each locality, the sporozoitic index and the EIR of *Anopheles gambiae*. Abdomens were also used for the molecular characterization using PCR for molecular forms [23] and species of the *An. gambiae* complex [24].

To assess the impact of LLINs (Olyset) on exophily and blood feeding of the vectors in R^+^ and R^+++^ areas, exit window traps and the morning pyrethrum spray catch were carried out inside the dwellings. Exit window traps used are similar to those described by Bar-Zeev and Self [25]. Thus, four exit window traps were set on plywood sheets that were fitted to window frames of the different dwellings for two nights each month. The selected dwellings contain a LLINs (Olyset) under which a person sleeps. Mosquitoes were collected from exit window traps the next day at 6 a.m. using a mouth aspirator. Then, resting mosquitoes inside these dwellings were collected on a white canvas after spraying aerosol Rambo^®^ inside dwellings from 7 a.m. to 8 a.m. Finally, the physiological state of abdomen of collected vectors by the two sampling methods was determined.

### Data analysis

The EIR representing the number of infected bites received by human per unit of time (night, month or year) was calculated for each locality and for each area in order to measure the intensity of malaria transmission. This rate was calculated by multiplying the Human Biting Rate (HBR) by sporozoitic index (S), (EIR = HBR × S).

The Poisson test [26] was used to compare the EIR between the different localities and also between R^+^ and R^+++^ areas. Infectivity, exophily, parity and blood feeding rates were compared between localities and, between the areas using Chi-square test or exact test of Fisher. Their confidence limit was determined using the binomial confidence limit method.

The factor that differentiates the 8 localities is the resistance level (R^+^ and R^+++^). However, the presence of permanent breeding sites in R^+^ area, precisely in Itassoumba (conversely in R^+++^ area) is a source of bias for some data, especially the EIR. For this reason, we used the multivariate generalized mixed model of Poisson [27] to evaluate the impact of resistance level of areas (R^+^ versus R^+++^) and the presence of permanent breeding sites (yes versus no) on the indicators of malaria transmission and of the behavior of vectors. This model was preferred based on AIC criterion (Akaike informative criterion) [28]. Data analysis was conducted using statistical package R, version R-2.15.2. [29].

### Ethical clearance

This study has been approved by the Ministry of Health of the Republic of Benin and its National Ethical committee for health research. The volunteer collectors of mosquitoes gave their consent before participating in the study. They were vaccinated against yellow fever and treated each time against malaria based on the Rapid Diagnostic Test of *P. falciparum.*

## Results

### Diversity of species of mosquitoes

Table 1 displays the different species of mosquitoes collected in both R^+^ and R^+++^ areas. Overall, the wildlife of Culicidae collected from human baits was more diversified regardless of the study areas. In total, 1989 mosquitoes were collected in R^+^ areas compared to 994 in R^+++^ areas. Otherwise, there were two times more mosquitoes in R^+^ area than in R^+++^ area. *Anopheles gambiae* was predominantly collected in each area, accounting for 75.51% of mosquitoes collected in the R^+^ area as compared to 56.94% in the R^+++^ area.

**Table 1:** Distribution of different species of mosquitoes per locality

	***R***^***+ ***^ ***localities***				***R***^***+++ ***^ ***localities***			
**Mosquitoes species**	**Itakpako**	**Itassoumba**	**Djohounkollé**	**Ko-Koumolou**	**Dagbao**	**Ossomou 1**	**Onigbolo**	**Idéna 2**
*Anopheles gambiae*	17	1455	16	14	24	12	382	148
*Anopheles funestus*	0	0	0	0	0	0	1	0
*Anopheles pharoensis*	0	1	0	4	0	1	3	0
*Anopheles ziemanni*	0	1	0	0	1	0	1	0
*Anopheles coustani*	0	1	0	0	0	0	0	0
*Aedes aegypti*	0	6	4	3	0	4	2	2
*Aedes vittatus*	0	0	169	4	1	0	5	0
*Aedes palpalis*	6	8	0	1	31	4	16	12
*Others Aedes*	0	0	0	0	1	0	0	0
*Culex quinquefasciatus*	25	9	85	10	51	24	57	10
*Culex gr decens*	3	2	0	0	2	1	0	0
*Culex nebulosus*	3	36	5	10	26	5	3	30
*Culex tigripes*	0	0	2	1	0	1	1	1
*Culex annulioris*	0	0	5	0	2	0	1	0
*Others Culex*	0	0	0	0	0	1	3	1
*Mansonia africana*	7	45	15	16	40	11	64	8
TOTAL/Locality	61	1564	301	63	179	64	539	212
TOTAL/Area	1989				994			

^+^: Low resistance; ^+++^: High resistance.

Besides the major malaria vector *An. gambiae*, another important vector *Anopheles funestus* was also collected in Onigbolo at a very low frequency. Other species of *Anopheles* collected were secondary malaria vectors including *Anopheles pharoensis, An. ziemanni,* and *An. coustani*. Their frequency was also very low (Table 1). Culicinae were less represented: 24.13% in R^+^ area against 42.35% in R^+++^ area. Culicinae constituted mosquitoes from the genus *Aedes, Culex* and *Mansonia. Cx. quiquefasciatus* and *Cx. nebulosus* were found in similar proportions in both areas. However, *Aedes vittatus* were abundant in R^+^ area (173 > 6) and *Mansonia africana* were more abundant in R^+++^ area (123 > 83). The predominance of one species of mosquito in one area in relation to other could be explained by the presence of favorable breeding sites for the development of this species in the aforementioned area. *Aedes aegypti* mosquitoes were collected in very low proportions in both areas.

### Aggressive density of *Anopheles gambiae* in R^+^ and R^+++^ areas

Table 2 shows the number of specimen of *An. gambiae* collected during the 8 captures organized on a monthly basis from July to November. Another collection of mosquitoes was carried out on 4 nights in December. During this study period, we collected 1502 *An. gambiae* from the inside and outside the dwellings of R^+^ area against 566 in R^+++^ area. In both areas, the biting behaviors of *An. gambiae* were similar. In both areas, the aggressiveness of these mosquitoes was more predominant on the outside (1087 specimens of *An. gambiae*) than the inside (415 specimens of *An. gambiae*) of dwellings particularly in R^+^ area (Table 2). Likewise, in R^+++^ area, the same observation was noted where 300 specimens of *An. gambiae* were collected on the outside compared to 266 on the inside of dwellings.

**Table 2:** Variation of vectors density inside and outside of dwellings

**Localities/areas**		**July-11**	**Aug-11**	**Sept-11**	**Oct-11**	**Nov-11**	**Dec-11**	**Total**
Itakpako	Indoor	3	4	0	0	2	0	9
	Outdoor	4	1	1	1	1	0	8
Itassoumba	Indoor	121	69	59	50	72	18	389
	Outdoor	177	170	75	121	405	118	1066
Djohounkollé	Indoor	1	2	0	4	3	0	10
	Outdoor	2	1	0	2	1	0	6
Ko Koumolou	Indoor	2	0	1	0	4	0	7
	Outdoor	5	1	0	1	0	0	7
*R*^*+*^*Area*	Indoor	127	75	60	54	81	18	415
	Outdoor	188	173	76	125	407	118	1087
Dagbao	Indoor	1	0	1	1	7	0	10
	Outdoor	0	0	1	3	9	1	14
Ossomou 1	Indoor	7	0	0	1	0	0	8
	Outdoor	3	0	0	0	1	0	4
Onigbolo	Indoor	100	6	2	50	2	0	160
	Outdoor	111	4	4	88	15	0	222
Idéna 2	Indoor	36	4	13	34	1	0	88
	Outdoor	10	6	7	35	2	0	60
*R*^*+++*^*Area*	Indoor	144	10	16	86	10	0	266
	Outdoor	124	10	12	126	27	1	300

^+^: Low resistance; ^+++^: High resistance.

### Variation of sporozoitic index in R^+^ and R^+++^ areas

Table 3 shows the number of mosquitoes analyzed by CSP ELISA and the number of mosquitoes that were found to be positive for each month. The monthly variation of infection rate of mosquitoes during the study period in each locality and in each area is indicated in the same table. Overall, in R^+^ area, 358 head-thoraxes of *An. gambiae* were examined of which 43 were positive for *Plasmodium falciparum* antigen with a mean sporozoïtic index of 12.01%. In R^+++^ area, 269 head-thoraxes were analyzed of which 31 were positive for *Plasmodium falciparum* antigen accounting for a mean sporozoïtic index of 11.52%. No significant difference was observed between the sporozoitic indexes for both areas (p = 0.85), suggesting that infection by mosquitoes is the same in both areas.

**Table 3:** Variation of sporozoitic index by locality and area

**Localities/areas**	**Parameters**	**July-11**	**Aug-11**	**Sept-11**	**Oct-11**	**Nov-11**	**Dec-11**	**Total**	**CI (95%)**
*R*^*+*^*Localities*									
Itakpako	N tested	5	1	0	1	2	0	9	
	Positive	0	0	0	1	0	0	1	
	S (%)	0	0	-	100	0	-	11.11^a^	[00.28-48.25]
Itassoumba	N tested	47	25	22	78	112	41	325	
	Positive	0	11	2	12	10	3	38	
	S (%)	0	44	9.09	15.38	8.93	7.32	11.69^a^	[08.41-15.69]
Djohoukollé	N tested	2	1	0	4	3	0	10	
	Positive	0	0	0	0	0	0	0	
	S (%)	0	0	-	0	0	-	0^a^	[00.00-30.90]
Ko-Koumolou	N tested	7	1	1	1	4	0	14	
	Positive	3	0	0	0	1	0	4	
	S (%)	42.86	0	0	0	25	-	28.57^a^	[08.39-58.11]
	N tested	61	28	23	84	121	41	358	
*R*^*+*^*Area*	Positive	3	11	2	13	11	3	43	
	S (%)	4.92	39.29	8.70	15.48	9.09	7.32	12.01	[08.83-15.84]
*R*^*+++*^*Localities*									
Dagbao	N tested	1	0	1	2	15	1	20	
	Positive	0	0	0	0	2	0	2	
	S (%)	0	-	0	0	13.33	0	10^a^	[01.23-31.70]
Ossomou 1	N tested	4	0	0	0	1	0	5	
	Positive	0	0	0	0	0	0	0	
	S (%)	0	-	-	-	0	-	0^a^	[00.00-52.20]
Onigbolo	N tested	53	9	6	68	10	0	146	
	Positive	1	0	0	13	0	0	14	
	S (%)	1.89	0	0	19.12	0	-	9.59^a^	[05.34-15.57]
Idéna 2	N tested	38	10	19	29	2	0	98	
	Positive	1	0	5	8	1	0	15	
	S (%)	2.63	0	26.32	27.59	50	-	15.31^a^	[08.83-23.99]
	N tested	96	19	26	99	28	1	269	
*R*^*+++*^*Area*	Positive	2	0	5	21	3	0	31	
	S (%)	2.08	0	19.23	21.21	10.71	0	11.52	[07.97-15.96]

S (%): sporozoitic index; ^a^Values sharing a superscript letter are not significantly different (p > 0.05); ^+^: Low resistance; ^+++^: High resistance.

### Variation of the EIR

Various categories of EIR were noted in the two areas. In R^+^ area, two situations were observed. Three localities overall yielded low values of EIR with a mean value of 7.73 infected bites (ib) in 6 months in Itakpako and 16.37 in Ko-Koumolou. However, in the locality of Djohounkollé, the EIR was null because no mosquito carried sporozoites out of the 10 specimens analyzed. In contrast to these three localities with low EIR, the fourth locality (Itassoumba) yielded a very high EIR with 695.95 infected bites in 6 months. The EIR are significantly different from one locality to the other in this area (p < 0.05).

The same observation was found in R^+++^ area. In the localities of Dagbao and Idena 2, individuals received mean values of 9.82 and 92.66 infected bites of *An. gambiae* respectively. However, in Onigbolo, the EIR was very high with 149.85 infected bites. Also, no anopheles mosquito was found to be infected in the locality of Ossoumou 1, which can be explained by the very low number of 5 mosquitoes captured during the collection period.

Overall, we note a higher transmission of malaria in R^+^ area (184.51 ib/h/6 months) than in R^+++^ area (66.7 ib/h/6 months) (p < 0.001) (Table 4).

**Table 4:** Variation of EIR by locality and area

**Localities/areas**	**Parameters**	**July-11**	**Aug-11**	**Sept-11**	**Oct-11**	**Nov-11**	**Dec-11**	**Total/period**
*R*^*+*^*Localities*								
	Total vector	7	5	1	1	3	0	17
	Man night	8	8	8	8	8	4	44
Itakpako	HBR/Night	0.88	0.63	0.13	0.13	0.38	0	0.38
	HBR/Period	26.25	18.75	3.75	3.75	11.25	0	69.54
	S (%)	0	0	-	100	0	-	11.11
	EIR/Périod	0	0	-	3.75	0	-	7.73^a^
	Total vector	298	239	134	171	477	136	1455
	Man night	8	8	8	8	8	4	44
Itassoumba	HBR/Night	37.25	29.88	16.75	21.38	59.63	34	33.06
	HBR/Period	1117.5	896.25	502.5	641.25	1788.75	1020	5952.27
	S (%)	0	44	9.09	15.38	8.93	7.32	11.69
	EIR/Périod	0	394.35	45.68	98.65	159.71	74.63	695.95^b^
	Total vector	3	3	0	6	4	0	16
	Man night	8	8	8	8	8	4	44
Djohounkollé	HBR/Night	0.38	0.38	0	0.75	0.5	0	0.36
	HBR/Period	11.25	11.25	0	22.5	15	0	65.45
	S (%)	0	0	-	0	0	-	0
	EIR/Périod	0	0	-	0	0	-	0^c^
	Total vector	7	1	1	1	4	0	14
	Man night	8	8	8	8	8	4	44
Ko-Koumolou	HBR/Night	0.88	0.13	0.13	0.13	0.5	0	0.32
	HBR/Period	26.25	3.75	3.75	3.75	15	0	57.27
	S (%)	42.86	0	0	0	25	-	28.57
	EIR/Périod	11.25	0	0	0	3.75	-	16.37^d^
	Total vector	315	248	136	179	488	136	1502
	Man night	32	32	32	32	32	16	176
*R*^*+*^*Area*	HBR/Night	9.84	7.75	4.25	5.59	15.25	8.5	8.53
	HBR/Period	295.31	232.5	127.5	167.81	457.5	255	1536.13
	S (%)	4.92	39.29	8.70	15.48	9.09	7.32	12.01
	EIR/Périod	14.52	91.34	11.09	25.97	41.59	18.66	184.51
*R*^*+++*^*Localities*								
	Total vector	1	0	2	4	16	1	24
	Man night	8	8	8	8	8	4	44
Dagbao	HBR/Night	0.13	0	0.25	0.5	2	0.25	0.54
	HBR /Period	3.75	0	7.5	15	60	7.5	98.18
	S (%)	0	-	0	0	13.33	0	10
	EIR/Périod	0	-	0	0	8	0	9.82^e^
	Total vector	10	0	0	1	1	0	12
	Man night	8	8	8	8	8	4	44
Ossomou 1	HBR/Night	1.25	0	0	0.13	0.13	0	0.27
	HBR/Period	37.5	0	0	3.75	3.75	0	49.09
	S (%)	0	-	-	-	0	-	0
	EIR/Périod	0	-	-	-	0	-	0^c^
	Total vector	211	10	6	138	17	0	382
	Man night	8	8	8	8	8	4	44
Onigbolo	HBR/Night	26.38	1.25	0.75	17.25	2.13	0	8.68
	HBR/Period	791.25	37.5	22.5	517.5	63.75	0	1562.72
	S (%)	1.89	0	0	19.12	0	-	9.59
	EIR/Périod	14.93	0	0	98.93	0	-	149.85^f^
	Total vector	46	10	20	69	3	0	148
	Man night	8	8	8	8	8	4	44
Idéna 2	HBR/Night	5.75	1.25	2.5	8.63	0.38	0	3.36
	HBR/Period	172.5	37.5	75	258.75	11.25	0	605.45
	S (%)	2.63	0	26.32	27.59	50	-	15.31
	EIR/Périod	4.54	0	19.74	71.38	5.63	-	92.66^g^
	Total vector	268	20	28	212	37	1	566
	Man night	32	32	32	32	32	16	176
*R*^*+++*^*Area*	HBR/Night	8.38	0.63	0.88	6.63	1.16	0.063	3.21
	HBR/Period	251.25	18.75	26.25	198.75	34.69	1.88	578.86
	S (%)	2.08	0	19.23	21.21	10.71	0	11.52
	EIR/Périod	5.23	0	5.05	42.16	3.72	0	66.7

^a,b,c,d,e,f,g^Values sharing a different superscript letter are significantly different (p < 0.05); ^+^: Low resistance; ^+++^: High resistance.

### The parity rate of anopheles

After each capture, *Anopheles* mosquitoes caught were dissected in order to determine the parity rate of the specimens. In R^+^ area, the parity rate varied from 66.66% in Itakpako to 84.61% in Ko-Koumolou. Out of a total of 698 *An. gambiae* dissected, 576 were parous yielding a parity rate of 82.52%. In R^+++^ area, a similar parity rate (79.27%) (241/304) was noted (p = 0.26) (Table 5). Moreover, comparing locality by locality reveals similar parity rates (p > 0.05).

**Table 5:** **Parity rate of *An. gambiae *** **by locality and area**

**Localities/areas**	**Total**	**Parous**	**Parity (%)**	**CI (95%)**
*R*^*+*^*Localities*				
Itakpako	9	6	66.66^a^	[29.93-92.52]
Itassoumba	650	539	82.92^a^	[79.80-85.74]
Djohounkollé	26	20	76.92^a^	[56.35-91.03]
Ko-Koumolou	13	11	84.61^a^	[54.55-98.08]
*R*^*+*^*Area*	698	576	82.52	[79.50-85.27]
*R*^*+++*^*Localities*				
Dagbao	23	20	86.95^a^	[66.41-97.23]
Ossomou 1	6	6	100^a^	[54.00-100.0]
Onigbolo	169	134	79.28^a^	[72.39-85.13]
Idéna 2	106	81	76.41^a^	[67.18-84.12]
*R*^*+++*^*Area*	304	241	79.27	[74.28-83.69]

^a^Values sharing a superscript letter are not significantly different (p > 0.05); ^+^: Low resistance; ^+++^: High resistance.

### Impact of LLINs on the behavior of *Anopheles* in R^+^ and R^+++^ areas

#### ***Level of induced exophily by the LLINs***

In R^+^ area, the exophily is 50% in Djohounkollé, 85.71% in Itakpako, 97.69% in Itassoumba and 78.57% in Ko-Koumolou; but in the R^+++^ area, this rate varied and ranged from 43.75% in Idéna 2 to 91.36% in Onigbolo. Overall, similar exophily rates were observed across 3 localities in the R^+^ area (Itakpako, Itassoumba and Ko-Koumolou) and 3 localities in the R^+++^ area (Dagbao, Ossomou 1 and Onigbolo) (Table 6). However, the cumulated data showed that the R^+^ area yielded the highest exophily rate (93.63%) compared to the R^+++^ area (73.65%) (p < 0.001).

**Table 6:** **Variation of *An. gambiae *** **exophiliy rate by locality and area**

**Localities/areas**	**Total**	**Exit**	**Exophily (%)**	**IC (95%)**
*R*^*+*^*Localities*				
Itakpako	7	6	85.71^a^	[42.13-99.64]
Itassoumba	432	422	97.68^a^	[95.78-98.88]
Djohounkolé	34	17	50^b^	[32.43-67.57]
Ko-Koumolou	14	11	78.57^ab^	[49.20-95.34]
*R*^*+*^*Area*	487	456	93.63	[91.09-95.63]
*R*^*+++*^*Localities*				
Dagbao	5	4	80^ab^	[28.36-99.50]
Ossomou 1	14	10	71.42^ab^	[41.89-91.61]
Onigbolo	81	74	91.35^a^	[83.00-96.46]
Idéna 2	48	21	43.75^b^	[29.48-58.82]
*R*^*+++*^*Area*	148	109	73.65	[65.78-80.54]

^a,b,ab^Values sharing a different superscript letter are significantly different (p < 0.05); ^+^: Low resistance; ^+++^: High resistance.

#### ***Blood feeding rate induced by the LLINs***

In the R^+^ area, out of a total of 487 of *An. gambiae* caught in the windows traps and in indoor spray catches, 160 females were blood fed generating a blood feeding rate of 32.85% (Table 7); but in the R^+++^ area, this rate was much higher at 42.66% (64/150) (p = 0.035). The proportion of unfed mosquitoes exiting houses and entering in window traps at Itassoumba was 69.7% (301/432) and that of fed mosquitoes reached 28% (121/432).

**Table 7:** **Variation of *An. gambiae *** **blood feeding rates by Locality and area**

**Localities/area**	**Total**	**Blood fed**	**Blood feeding (%)**	**CI (95%)**
*R*^*+*^*Localities*				
Itakpako	7	1	14.28^a^	[00.36-57.87]
Itassoumba	432	121	28^a^	[23.82-32.50]
Djohounkollé	34	27	79.41^b^	[62.10-91.30]
Ko-Koumolou	14	11	78.57^b^	[49.20-95.34]
*R*^*+*^*Area*	487	160	32.85	[28.69-37.22]
*R*^*+++*^*Localities*				
Dagbao	3	1	33.33^ab^	[00.84-90.57]
Ossomou 1	14	11	78.57^b^	[49.20-95.34]
Onigbolo	83	14	16.86^a^	[09.54-26.68]
Idéna 2	50	38	76^b^	[61.83-86.94]
*R*^*+++*^*Area*	150	64	42.66	[34.63-50.99]

^a,b,ab^Values sharing a different superscript letter are significantly different (p < 0.05); ^+^: Low resistance; ^+++^: High resistance.

### Results of multivariate analysis

#### ***Impact of the level of resistance on the EIR***

Table 8 displays the impact level of resistance of *Anopheles* for the two areas of resistance and the permanent presence of *Anopheles* breeding sites on the EIR. The risk for a human to receive the infected bites of *Anopheles* in the R^+++^ area is 2.71 times higher than in the R^+^ area. Nevertheless, this relative high risk is not linked to the R^+++^ resistance status of the *Anopheles* mosquitoes for this area but instead to the fluctuation of the sample. On the other hand, results from statistical analysis reveal that, there is no impact of the resistance level of *Anopheles gambiae s.l.* on the transmission of malaria (p = 0.43).

**Table 8:** Analysis of the impact of the level of resistance and the presence of permanent breeding sites on the EIR

**Parameter**	**Sources of variation**	**Modalities**	**Coef**	**OR**	**CI-95%**	**p (> Chisq)**
EIR	Areas	R^+^	0.000	1.00	-	0.43
		R^+++^	0.996	2.71	[00.22-12.44]	
	PBS	A	0.000	1.00	-	< 0.001
		P	1.013	2.75	[02.55-02.97]	

PBS: Permanent breeding sites; P: Presence; A: Absence; ^+^: Low resistance; ^+++^: High resistance.

The presence of permanent mosquitoes breeding sites increases the risk of human receiving infected bites from *Anopheles* mosquitoes by 2.75 fold (Table 8). Furthermore, the results showed that the presence of permanent breeding sites strongly influences the EIR (p < 0.001).

#### ***Impact of resistance level on the behavior of anopheles***

Table 9 shows that the probability of *Anopheles* vectors that are blood-fed is 1.27 times higher in the R^+^ area [OR (R^+^) = 1.00, OR (R^+++^) = 0.79]. This unexpected result is not due to the level of resistance of *Anopheles* (p = 0.35), but due to the fluctuation of the sample. Consequently, the presence of permanent breeding sites for mosquitoes strongly impacts the blood-feeding rate (p = 0.01). In the presence of permanent breeding sites, vectors are two times less likely to take their blood meal [OR (PBSA) = 1.00, OR (PBSP) = 0.54].

**Table 9:** Analysis of the impact of the level of resistance and the presence of permanent breeding sites on the blood feeding and the exophily rates

**Parameters**	**Sources of variation**	**Modalities**	**Coef**	**OR**	**CI-95%**	**p (> Chisq)**
Blood feeding	Areas	R^+^	0.000	1.00	-	0.35
		R^+++^	-0.227	0.79	[00.49-01.28]	
	PBS	A	0.000	1.00	-	0.01
		P	-0.609	0.54	[00.34-00.87]	
Exophily	Areas	R^+^	0.000	1.00	-	0.14
		R^+++^	-0.232	0.79	[00.46-01.37]	
	PBS	A	0.000	1.00	-	0.02
		P	0.647	1.90	[00.85-04.31]	

PBS: Permanent breeding sites; P: Presence; A : Absence; ^+^: Low resistance;^+++^: High resistance.

The probability that malaria vectors will exit houses is 1.27 times stronger in the R^+^ area [OR (R^+^) = 1.00, OR (R^+++^) = 0.79] and, the presence of permanent breeding sites increases this probability by 1.9 [OR (PBSP) = 1.90, OR (PBSA) = 1.00] (Table 9). Indeed, the results of the analysis have shown that the exit of *Anopheles* from houses is not under the influence of their resistance level (p = 0.14). It is rather influenced by the presence of permanent breeding sites that allow the population of *Anopheles* vectors to increase (p = 0.02).

## Discussion

We observed a great diversity of Culicidae species that were collected in the two areas of resistance. The results obtained reveal that *An. gambiae s.l.* is the major malaria vector in both areas of resistance.

Considering that the study period overlaps with the rainy season, we might expect a very high anopheline density inside human dwellings given that this is the time of year where people sleep indoors; but this was not observed during our study. Indeed, during the rainy season, it is less hot and, people rarely sleep outside of their houses. Therefore, there is no reason for *An. gambiae* that is usually indoor host seeking, to bite more on the outside than on the inside of dwellings. The low density of *An. gambiae* collected inside could be related to the excito-repellency effect of permethrin used to impregnate the nets (Olyset). Similar results were obtained by Reddy *et al*. [30] who showed that *An. gambiae* looked for their hosts outside houses, after an indoor residual spraying campaign that was combined with the distribution of LLINs in the island of Bioko (Equatorial Guinea).

Our results reveal a stronger aggressive density of *An. gambiae* in the R^+^ area than in the R^+++^ area (p < 0.001). This could be explained by the fact that Itassoumba, one of the R^+^ localities is remarkably characterized by a higher number of mosquitoes (*An. gambiae*)*,* due to its location near a market garden with several fishpond basins. Manga *et al*. [31] who conducted studies in Cameroon, Klinkenberg *et al.*[32] in Ghana and Yadouléton *et al*. [33] in Benin have shown that the market gardens are the areas for strong development of mosquito larvae. Gardeners and pisciculturists constantly maintain small basins of water collections for watering plants and breeding of fishes respectively. These water collections constitute the quasi-permanent breeding sites for *Anopheles* larvae irrespective of the season in the year. Gardeners also use fertilizers to boost the productivity of their vegetables. A study conducted in 2009 by Dadzié (personal communication) in controlled laboratory conditions have shown that fertilizers applied at sub-lethal doses to larvae could reduce the duration of their development cycle and significantly increases the emergence of adult mosquitoes. The shortening of the development cycle marked with an increase of the emergence rate induced by the presence of these organic substances at sub-lethal doses could increase the density of vectors. This could lead to an increase in the risk of malaria transmission even if other parameters like age or trophic preferences of vectors are considered. Therefore, it is possible that the relation between the use of fertilizers and the increase in vectorial density observed under laboratory conditions is also possible in the market gardening and fishpond perimeters of Itassoumba. Likewise, the expected predatory behaviour of the breeding fishes was unnoticeable because of the high anopheline density recorded in this location. In turn, *An. gambiae* larvae fed on the same feed grains used for fish farming. Thus, unlike Itassomba, *An. gambiae s.l.* breeding sites were rare in the other localities (Itakpako, Djohounkollé, Ko-Koumolou, Dagbao, Ossomou 1, Onigbolo and Idena 2) during the dry season. Rather, we noted the permanent presence and the abundance of *An. gambiae* in Itassoumba. Looking at the data of all localities by area of resistance, higher biting rates in the R^+^ area than in the R^+++^ area were noticed.

Our results showed no significant difference between the infectivity of *An. gambiae* population to *Plasmodium falciparum* in both areas. In the R^+^ area, the sporozoitic index is 12.01% against 11.52% in the R^+++^ areas (p = 0.85). But the higher EIR observed in the R^+^ area (184.51 ib/h/6 months) compared to the R^+++^ area (66.7 ib/h/6 months) (p < 0.001) is especially due to the higher aggressive density of *An. gambiae* recorded in Itassoumba. This hypothesis is supported by the multivariate analysis, which showed that the level of resistance of malaria vectors in each area does not influence the level of transmission. However, the presence of permanent breeding sites of *An. gambiae* in Itassoumba (R^+^ locality) has a significant impact on the EIR observed in the R^+^ area.

Taking into account the disparity in the distribution of the breeding sites of *Anopheles* mosquitoes, we question whether EIR is the best parameter of the measure of the impact of malaria vectors’ resistance to pyrethroids on the effectiveness of LLINs. Though in the formula EIR = HBR × S, “HBR” constitutes a variable that depends on the environment which might be reduced by the use of impregnated bed nets, and thus highly influences the value of EIR. For this reason, EIR remained high in Itassoumba because of the “HBR” which was extremely high. On the other hand, sporozoitic index seems to be a better parameter because it is directly linked to the intrinsic behavior of *An. gambiae* in the environment.

The different levels of malaria transmission observed in the two areas (R^+^ and R^+++^) are very high in spite of the presence of Olyset Nets. According to Trape *et al.*[34], control interventions will help to eradicate malaria only if they are able to reduce personal exposure to less than two infected bites of *An. gambiae* per human per year. In other words, this requires for instance a reduction of more than 99% in the level of transmission in an R^+^ area. In order to achieve this goal to eliminate malaria, a good coverage in LLINs is not only required, but also their appropriate use and the management of the environment combined with curative measures are required. Also, to reach an optimal efficacy of the LLINs under field conditions, a universal and complete coverage of people living in endemic area is recommended by the World Health Organization [35]. According to the same source, a coverage rate in LLINs over 80% helps reduce mortality rates in children and youth to at least 25%. Furthermore, according to a recent study conducted in the south coast of Kenya, a drastic decrease of more than 99% of the EIR (20.44 ib/man in 1997–1998 to 0.15 ib/man in 2009–2010) in *An. gambiae* has been observed after 13 years of utilization of LLINs with a coverage rate of more than 86% and a mean of 1 net for 2.5 persons [36]. Moreover, Akogbéto *et al*. [37] also reported a reduction in EIR of more than 70% in the district of Dangbo in Southern Benin following a massive use of LLINs.

According to Gnanguènon *et al.*[38], resistant mosquitoes which could stand the excito-repellency effect of LLINs, have the ability to penetrate impregnated bed nets and thus feed on humans. However, the absence of blood meal affects the mosquito’s longevity depriving it from a potential source of energy indispensable for its development. Thus, it is not excluded that highly resistant mosquitoes are likely to live longer than the susceptible or less resistant ones. But, in the current study, no significant difference was observed between the parity rates of *An. gambiae* collected in the R^+^ area and in the R^+++^ area (p = 0.26). This could be explained by the fact that there is a weak difference between low resistant mosquitoes and high resistant mosquitoes that were compared (Djègbè, personal communication). In any case, low resistant mosquitoes could not be considered as a susceptible strain.

The high exophily observed in the R^+^ area as opposed to the R^+++^ area (p < 0.001) is not solely due to the excito-repellency effect of impregnated nets, but it is also due to the fact that in this area, and particularly in Itassoumba, *Anopheles* density inside houses is very high because of the abundance of *Anopheles* in the environment. In this context, a great part of the mosquitoes seeking hosts inside houses could be constrained to go out to seek other hosts as a result of an unsuccessful blood intake.

The low blood feeding rate of *Anopheles* recorded in the R^+^ area compared to the R^+++^ area (p = 0.035) confirms the hypothesis that when the density of *Anopheles* inside houses is high as in Itassoumba, most of the mosquitoes fail to have their blood meal. This supports the low blood feeding rates recorded in the R^+^ area since the unfed mosquitoes seek for their hosts outdoors. This hypothesis is further supported by the multivariate analysis, which revealed that the level of resistance of *Anopheles* in both areas does not influence their aptitude to take the blood meal or to exit houses. On the other hand, the presence of permanent breeding sites with highly productive *An. gambiae* strongly affects these two parameters.

To really evaluate the impact of the resistance of malaria vectors to pyrethroids on the effectiveness of LLINs, it would be desirable to have two areas: one where the *Anopheles* are resistant and another one, where *Anopheles* populations are fully susceptible. In addition, the two areas must have the same ecological patterns. Unfortunately, we could not find such areas and this constitutes the main limitation of this study. Another limitation of this study is that, we have not taken into account a confounding factor such as the use of the LLINs in the two resistance areas. It would also be interesting to do a tunnel test using the LLINs, with field collected mosquitoes from R^+^ and R^+++^ areas. It would have shown if the R^+^ and R^+++^ mosquitos can penetrate the LLINs and fed on the mice on the other side.

## Conclusion

The main goal of this study is to determine if the resistance of malaria vectors to pyrethroids affects the efficacy of LLINs. After tracking the behavior of anopheles in contact with Olyset nets for 6 months in the area where *Anopheles* population is highly resistant (R^+++^) and in the area where it is less resistant (R^+^), the entomological indicators of the transmission (parity rate, sporozoitic index) were similar in both areas. The susceptibility of *An. gambiae* populations is the same for *Plasmodium falciparum* in R^+^ and R^+++^ areas. The high EIR observed in R^+^ area is mainly due to the aggressive density of *An. gambiae*, which was particularly high in Itassoumba. This observation is confirmed by the multivariate analysis which confirmed that the resistance level of the vectors in each area does not influence the level of transmission of the parasite. Furthermore, the higher rate of the exophily and the low blood feeding rate observed in the R^+^ areas compared to the R^+++^ areas is not related to the resistance status of *An. gambiae*, but instead, to the differences between both areas in terms of distribution and the availability of *Anopheles* breeding sites. This phenomenon is not related to malaria vectors’ resistance but to the environment. As a result, in our study, the impact of resistance on behavior and on the effectiveness of LLINs in the reduction of malaria transmission was not observed.

## Competing interests

The authors declare that they have no competing interests.

## Authors’ contributions

AS, RA, RYA, RG, FT and MA conceived the study. AS, MO, BA, DG, AM and MA have participated in the design of the study. AS, ASS, RG and FA carried out the field activities and the laboratory analysis. FOA has contributed to the data analysis. AS and MA drafted the manuscript. MA, AM, RA, RYA, FT and AS critically revised the manuscript for intellectual content. All authors read and approved the final manuscript.
